# Analyzing Consumer Protection for Gamblers Across Different Online Gambling Operators: A Replication Study

**DOI:** 10.1007/s11469-021-00695-9

**Published:** 2021-11-09

**Authors:** Maris Catania, Mark D. Griffiths

**Affiliations:** 1 Kindred Group, Tigne Point, Level 6, The Centre, Sliema, TPO001 Malta; 2 International Gaming Research Unit, Psychology Department, Nottingham Trent University, 50 Shakespeare Street, Nottingham, NG1 4FQ UK

**Keywords:** Gambling operators, Responsible gambling, Player protection, Harm-minimization, Social responsibility, Consumer protection

## Abstract

Online gambling is a growing business with many stakeholders. Due to the fact that a small proportion of gamblers develop problems, responsible gambling (RG), player protection, and harm minimization have become core areas for gambling regulators. The present study replicated a previous one carried out by Bonello and Griffiths in 2017 to determine whether there had been any significant changes by leading gambling operators due to increased regulatory pressures over the past few years. Fifty leading online gambling operators were audited in relation to their RG practices as well as engaging with their customer services by posing as a problem gambler. Results indicated that overall RG practices appeared to have improved in the past 3 years based on the information in dedicated RG webpages, the increase in RG tool availability, and the communication with customer services. Despite the fact that RG practices appear to have improved, there were still areas for improvement.

Online gambling is a rapidly growing e-commerce sector whereby individuals can gamble via their mobile phones, computers, and wireless devices (Gainsbury et al., 2012). Consumers are now able to gamble almost anywhere due to technological advancements via smartphones and the internet (Auer & Griffiths, 2013). Online gambling has many different stakeholders including gamblers, health services, gambling industry, regulators, and the government (Blaszczynski et al., 2004). More recently, the gambling industry has introduced responsible gambling (RG) initiatives to help minimize the harm that gambling may be causing.

Responsible gambling and the message to “gamble responsibly” have been commonly used by the industry in their communication channels and in the promotional material on the gambling services that they offer (Hing et al., 2018). RG refers to practices that are employed in order to reduce and potentially prevent harms associated with gambling (Blaszczynski et al., 2004). Gambling operators echo this in their practice but also mention that RG is a shared responsibility, and in some instances, it is highlighted that the main responsibility lies with the consumer (Forsstrom & Ornberg, 2019).

Some claim that RG policies should be enforced through a tripartite model which is not limited to the gambling operators and the individual but also the government (Blaszczynski et al., 2011). Gambling regulation is usually centered around the possible risks associated with gambling and with the main role being consumer protection (Villeneuve & Meyer, 2010). Despite this, in some jurisdictions such as Malta, online gambling may be more viewed as an economic issue rather than around its potentiality of being a health problem (Marinaci et al., 2019). Online gambling has gone through an overhaul in terms of consumer protection in the past 5 years due to an increasing amount of countries regulating online gambling and in turn enforcing consumer protection measures. It is one thing to have such regulations in place, but it is another to have them in practice. In fact, such discrepancies have resulted in penalties and in a few instances gambling licenses being revoked. For instance, in 2018, the UK Gambling Commission threatened to revoke five online gambling operator licenses due to failures in consumer protection in the prevention of anti-money laundering and consumer interactions (Kott, 2020a).

Penalty examples include a gambling operator being fined £2.3 million for accepting transactions from consumers who gambled large amounts of money, which were only possible by stealing (Gambling Commission, 2017). Another gambling operator was fined £13 million due to money laundering offenses as well as social responsibility failures, which resulted in senior managers from the online gambling company losing their personal gambling license (Gambling Commission, 2020a). These penalties were not only restricted to financial fines but also (in some cases) having the operator’s license suspended (Gambling Commission, 2020b).

This increased focus on RG has also been seen in other gambling jurisdictions, such as Belgium, where a draft law was submitted at the beginning of 2020 to increase proactivity in the prevention of problem gambling. Here, operators are obliged to not only inform consumers about gambling risks but also take a proactive role in helping individuals to moderate their gambling consumption (Kashina, 2020a). Denmark updated their secondary gambling legislation so to increase consumer protection to continue enforcing RG practices (Kott, 2020b). On the first day of 2019, a new licensing regime was introduced for Swedish operators that included RG measures such as RG-related messaging when the consumer accesses their gambling account, along with information about RG tools, and their accrued total financial losses for the previous year (Kott, 2020c). Spelinspektionen (the Swedish gambling regulator) followed on the footsteps of the UK Gambling Commission and introduced financial penalties for operators failing RG measures, as well as having the power to revoke gambling licenses (Altaner, 2020). This increased RG focus has also had its effect on the Maltese regulator where new regulations were proposed in 2018 which included an increase in RG messaging to its consumers (Kashina, 2020b).

In order to better understand gambling operators’ practices, researchers have attempted to explore this practice through descriptive studies. Smeaton and Griffiths (2004) were the first to do this by examining the RG practices of 30 online gambling websites based in the UK. It was evident that more attention was needed in this area as there was a lack of RG initiatives at the time (Blaszczynski et al., 2004). Smeaton and Griffiths reported that 23 out of the 30 examined operators had no reference to responsible gambling, and 26 out of the 30 operators did not have a reference or referral to a gambling help organization. This lack of RG commitment was further reflected by the fact that only one of the operators examined had an option to self-exclude. Marionneau and Jarvinen-Tassopoulos (2017) carried out a similar study of French online gambling operators where it was noted that RG tools were not always easy to use, and information about RG was not on the same platform that was used to gamble on. However, it was noted that every licensed website examined conformed to all the RG obligations placed by the law, including having a warning banner, an option to self-exclude, an option to set up limits, and information based on the consumer’s account history.

Bonello and Griffiths (2017) evaluated the 50 most advertised gambling operators in Malta to understand the different consumer protection practices available to gambling consumers. Their study showed that the majority of operators had an RG page, but most of these pages contained commercial information on this page. Most operators offered the possibility to set limits and to self-exclude. Cooney et al. (2021) evaluated online gambling operators based in Ireland (replicating the study by Bonello and Griffiths [2017]) and concluded that RG tools were inconsistent. The majority of the operators had an RG page and included links to gambling help organizations. Limits were available across most of the operators, almost half offered session-time reminders, and almost all offered self-exclusion options, through customer service or online application. Consistency across different operators would help as most gamblers use more than one online gambling account (Auer & Griffiths, 2012), and therefore having consistent RG tools being used by different gambling operators would help in minimizing harm. Moreover, RG tools not only increase customer trust, but also result in less disputes due to gambling issues (Gainsbury et al., 2013).

## The Efficacy of Responsible Gambling Tools

There have been a number of reviews concerning the efficacy of responsible gambling tools over the past few years including both general reviews of all RG tools (e.g., Drawson et al., 2017; Harris & Griffiths, 2017; Ladouceur et al., 2017; McMahon et al., 2019; Tanner et al., 2017) and reviews of very specific RG tools such as pop-up messaging (Bjørseth et al., 2021) and limit-setting (Delfabbro & King, 2021).

Ladouceur et al. (2017) systematically reviewed all studies that had examined the efficacy of RG tools that had been examined in a real-world gambling environment. The review included 29 studies and primarily covered self-exclusion programs and limit-setting tools with only six studies providing the “scientific rigor necessary to offer interpretative confidence,” and the evidence concerning efficacy of RG tools and programs was “very limited” (p.233). Harris and Griffiths (2017) examined the efficacy of RG tools that were used by the gambling industry to reduce gambling-related harm (i.e., mandatory play breaks, limit-setting, pop-up messaging, and behavioral tracking tools). They evaluated 20 studies and noted many of the methodological weaknesses of the studies examined. While there appeared to be some efficacy in relation to limit-setting, the efficacy of other RG tools was somewhat limited.

Drawson et al. (2017) examined the efficacy of what they described as “protective behavioral strategies in gambling” (e.g., monetary limit-setting, time limit-setting, self-exclusion). They reviewed 33 studies and concluded that evidence was best for self-exclusion programs but that the findings across all studies was inconsistent and that there were many methodological limitations that affected the generalizability of the results. The same team carried out a systematic review of gambling industry-implemented harm-minimization strategies (Tanner et al., 2017). They reviewed 27 studies and concluded that there was some preliminary efficacy among RG tools such as self-appraisal pop-up messages, removal of large-note acceptors on slot machines, and $1 maximum bets. However, the quality of the studies was limited, and there was a reliance on retrospective self-report alongside the lack of adequate control groups.

McMahon et al. (2019) carried out a review of systematic reviews and examined 55 studies within 10 systematic reviews examining RG tools such as self-exclusion, personalized messaging, pre-commitment, and limit setting. They concluded that the efficacy of such tools was limited by the extent to which gamblers adhere to voluntary (rather than mandatory) systems. They also claimed that the studies evaluated were “generally poor” and “dominated by evaluations of individual-level harm reduction interventions, with a paucity of research on supply reduction interventions” (p.380).

Bjørseth et al. (2021) carried out a meta-analysis examining the efficacy of pop-up messaging in 18 published studies although only three of these were real-world studies with most carried out in laboratory settings. They concluded that pop-up messaging had moderate effects in curtailing gamblers’ behavior and that pop-ups are an important part of gambling operators’ RG tool portfolio. Delfabbro and King (2021) carried out a systematic review of mandatory and voluntary pre-commitment and limit-setting RG tools. The review examined 25 studies and expressed caution about the potential benefits of limit-setting systems, but some mandatory limit-setting tools appeared to have at least some efficacy.

### The Present Study

Given the relative lack of research and that the RG demands by regulators are increasing, the aim of this present study was to replicate the study of Bonello and Griffiths (2017) and to examine whether there were any notable changes as a result of the increased regulatory pressures. An assumption was that operators would have increased their RG efforts, especially since most online operators are operating in a multi-license environment. Details of the specific RG practices that were investigated are found in the following section. The study examined best international practices regarding RG. Given that many operators provide gambling services to multiple countries, and different countries have different gambling regulations, the authors took the approach of examining what RG tools were utilized by operators rather than trying to determine whether the operator adhered to specific country regulations regarding harm minimization.

## Method

The present study replicated Bonello and Griffiths’ (2017) study where the 50 most popular online gambling websites were examined. The original aim of the study was to evaluate exactly the same gambling operators that were examined as those in the study of Bonello and Griffiths (2017) because this was a replication study. However, 18 of these gambling operators were no longer in operation, and one operator was not evaluated because one of the authors is employed by the parent company of one of the gambling operators included in the previous study. Therefore, 19 new gambling operators were included in the present study. The selection of the newly added operators was based on online search engines where the most advertised online gambling websites with a .com suffix were chosen. All the online gambling websites used in the study were licensed. Each online gambling operator was examined in detail by inspecting the following RG practices, which are an exact replication of the Bonello and Griffiths study conducted in 2017:

- A dedicated responsible gambling page containing the following:
  - An account of the operator’s commitment to responsible gambling
  - A warning that gambling may be harmful
  - Point of reference to organizations that can provide problem gambling assistance and help
  - Self-assessment test to help awareness of an individual's potential problem gambling
  - Information on the responsible gambling tools that the operator has in place
  - No promotional or enticing gambling material
  - Links to gambling filtering software including examples such as *Gamban* and/or *Betfilter*
- Age checks and warnings about underage play at account registration
- Responsible gambling information in the email communication sent to the online consumer
- The presence and accessibility of the gambler’s account history
- The availability of responsible gambling tools, such as:
  - Means for setting limits
  - Cooling-off periods or more popularly known as “take a break”
  - Self-exclusion possibilities
  - Other responsible gambling tools
- Customer service communication showing a commitment to responsible gambling

With regard to evaluating the customer service communication, contact was made with the customer service representatives. The questions and statement used to assess this were identical to the previous study by Bonello and Griffiths (2017):

1. “*I would like to control my gambling. Do you have any information on how I can do that?*”
2. “*What happens if I increase or remove any of the limits I set?*”
3. “*I feel addicted sometimes and cannot control my gambling.*”

As in the previous study, this aforementioned communication was explored via live chat, and where this possibility was not present, e-mail was used. Due to the method employed, full transcripts of the online conversations were obtained.

### Data Analysis

Data analysis comprised two different elements. For analysis of the RG page content and what RG tools were offered by the gambling operator, a basic content analysis was performed to see what information was provided on these pages and which RG tools the operators utilized on their website. In order to collect the data, the first author created an online gambling account with all 50 online gambling website. A pseudonym was created for the account, as well as a fictional date of birth and address. A dedicated email was created for this study, and a real phone number was used in case customer services wanted to contact the first author. With regard to the customer service interaction, the replies were grouped in the following manner. For the first question, if the response by customer services consisted of promoting RG practice or tools, this was rated as positive. For the second question, the response expected by customer services was that there should be an increase or removal of such limits and resulting in a “cooling off” period. If this happened, it was assessed positively. For the final statement, the responses by customer services were categorized as (i) good practice, (ii) terminated account, and (iii) bad practice. Good practice comprised providing assistance, RG information, and information on how to seek help for a gambling problem. Terminated account referred to when the operator did not initiate communication about RG and help-seeking but simply terminated the relationship with the customer without the customer being told anything by the operator. Bad practice comprised any operator that did not provide help and information or terminate the account.

## Results

In some instances, it was found that a number of online gambling operators had a strong commitment to responsible gambling, but other online gambling operators showed much less when comparing these online gambling websites with each other, even from a compliance perspective. An evaluation of each of the responsible gambling practices mentioned in the previous section is described below.

### Responsible Gambling Page

Not all online gambling operators had an RG page, and therefore the results below are based on 48 (rather than 50) operators. Out of the operators that had an RG page, all had a statement about their commitment towards RG and a warning that gambling may cause problems or negative consequences. Most operators (*n* = 46; 92%) referenced a help group where the consumer may seek help with problem gambling, and 42 had a self-assessment test available (84%). Eight operators used the Problem Gambling Severity Index (PGSI; Ferris & Wynne, 2001) as a self-assessment tool, and one operator had adapted the DSM-5 criteria for gambling disorder (American Psychiatric Association, 2013). Four operators offered a link to the Brief Biosocial Gambling Screen (BBGS; Gebauer et al., 2010). Four operators used the GA-20 Test (Gamblers Anonymous, not dated). Eight operators provided a link to external websites where gamblers could take a self-test. More specifically, four operators provided a link to the UK charity *GamCare* where the customer was then redirected to a link for the self-assessment test developed by *Sustainable Interaction*. Three operators provided a direct link to the self-assessment test developed by *Sustainable Interaction*, and one operator provided a link to *Gamblers Anonymous* website. Ten operators provided a range of different problem gambling self-assessment tests which did not appear to be based on psychometrically valid problem gambling assessment screens.

Information about RG tools was available on the RG page for 46 operators (92%) although some of the information was misleading, or too technical and provided legal information. An example of where an operator gave misleading information was where there was a mention that the consumer could self-exclude online by clicking on the link provided, but the link redirected the consumer to a contact page. Another example included an operator’s RG page which was full of information about different RG tools, but the only RG tool available was session limits to be set by the consumer. Most operators (*n* = 46) did not display any commercial information on the RG page (92%), but the few that did were “aggressive” in doing this via several pop-ups. However, this was only visible once the consumer was logged in. More than half of the operators (*n* = 27) displayed information or a link to gambling filtering software (54%).

### Age Verification

When the first author registered the gambling account, the majority of the online operators (*n* = 44; 88%) made it clear to customers that the gambling service was available only to individuals over the age of 18 years. However, in no instance was the first author asked to provide evidence of any form of identification document.

## Responsible Gambling Information Sent via E-mail

Three-quarters of the operators (*n* = 37; 74%) sent an e-mail to the consumer as soon as the registration was completed. These emails were mainly of a commercial nature to encourage the consumer to gamble, to benefit from a bonus, or “free” money to gamble. Only 21 of the 37 operators had any RG information or a link to the RG page. Four operators provided a link to an RG page where the link was, and in one instance, it directed the consumer to the “gambling bonus” page instead, which may have been on purpose or may have been a technical fault. As noted in more detail below, the first author communicated with the customer service agent and explicitly mentioned that they had a possible gambling addiction. A number of gambling regulators specifically state that any commercial communication must not be directed to known vulnerable individuals such as those who have self-excluded or have let the gambling operator know they have a gambling problem (e.g., Malta Gaming Authority, 2018). This means that the online operator should refrain from sending promotional communication to such consumers, and most of the operators did this (*n* = 43; 86%). However, seven operators continually sent promotional communication following self-exclusion. The highest occurrence of promotional communication was five emails in 14 days, and out of these seven operators, three of them also sent promotional bonus material to the first author’s smartphone in the form of a text message.

### Access to Gambling Account History

Almost all the operators (*n* = 48; 96%) provided an easy and clearly accessible option for the consumer to gain access to their gambling transactions.

### Responsible Gambling Tools

All but one of the operators (*n* = 49; 98%) provided the consumer the possibility to set up a limit, and 48 of the operators (96%) offered the options to take a break or to self-exclude. The RG tools were not consistent among the operators evaluated, and the availability of tools is outlined in Table 1.

**Table 1:** Types of RG tools offered by online gambling operators

RG tool	RG tool definition	Occurrence
Deposit limit	Option to limit how much money can be deposited daily, weekly, and/or monthly	*n* = 39; 78%
Loss limit	Option to limit monetary losses per day, week and/or month	*n* = 19; 38%
Time limit	Option to limit how much time is spent on the website either per session or over a specific time period such as daily, weekly, and/or monthly	*n* = 16; 32%
Reality check	Option to set a reminder every x minutes which would then prompt whether or not to continue gambling	*n* = 15; 30%
Wager limit	Option to set a limit on the amount of money wagered on a single instance	*n* = 13; 26%
Spending limit	Option to set a limit on how much money is spent daily, weekly, and/or monthly	*n* = 2; 4%
Activity statement	Option to have a statement showing financial transactions for a chosen period	*n* = 2; 4%
Product block	Option to block access from a specific type of gambling for a designated period of time	*n* = 2; 4%
Turnover limit	Option to set a limit on financial turnover	*n* = 1; 2%
Budget calculator	Option to input salary figures to suggest the amount of money that can be afforded to spend gambling	*n* = 1; 2%

Cooling-off periods (sometimes referred to as the “take a break” option) are the options that allow customers to pause their gambling for a period of less than 6 months. This facility was available among most operators (*n* = 48; 96%). Voluntary self-exclusion option, which refers to taking a break from gambling for 6 months or more, was a facility also offered by most operators (*n* = 48; 96%). Although these options were said to be available, the information provided to the consumer was not always truthful in a minority of instances. There were two operators where these tools could not be accessed. In one instance, the consumer had to deposit money into the account, and for another operator, the consumer had to accept and claim a bonus. In another instance, the RG information or link to the RG page was not available on the main page where all other features, including depositing money and playing a multitude of games, were available. One operator claimed that they had many RG tools available, but only a reality check option was available. One operator required the completion of a self-assessment test prior to accessing the RG tools, which although might have good intentions might discourage the gambler because it may be viewed as an extra unnecessary hurdle.

### Interactions with Customer Service

To better understand properly RG practice available to the consumer, two questions and one statement were presented to the customer service team (see the “Methods” section). The best method to speak to the customer service representative is by communicating with the operator via a live chat facility, and if this is unavailable, via email. Most operators had a live chat facility (*n* = 42; 84%); therefore, only eight operators needed to be contacted via email.

The results are presented for answers given either by live chat or email. Seven operators out of fifty declined to answer any of the first author’s questions. After repeated attempts, no answers were provided. With regard to the first question asked (“*I would like to control my gambling. Do you have any information on how I can do that?*”), all the remaining operators replied in a good RG-oriented manner, mentioning and advocating the use of limit-setting. The extent of the reply varied from just mentioning the deposit limit to other operators going as far as also sending information about treatment centers. The second question provided many different answers (“*What happens if I increase or remove any of the limits I would set?*”). The majority of the operators (*n* = 39; 78%) replied in a positive and helpful way by indicating that an increase of the limits would essentially mean that the consumer would need to wait for 24 hours (and one operator mentioned 7 days). Three of the operators did not really answer the question, and this appeared to be because there was a lack of knowledge from the customer service representative.

The last statement presented to the customer service agent was “I feel addicted sometimes and cannot control my gambling.” In order to evaluate the answers for this statement, the responses were categorized in three ways: (i) suggestions that would qualify as good practice (*n* = 19; 38%), (ii) action taken on the account without informing the consumer (*n* = 12; 24%), and (iii) bad practice (*n* = 11; 22%). The operators that fall under the first category of good practice gave the first author suggestions on how to handle potential gambling problems and also sent information about where to seek help. It was notable that the suggestions were done in a friendly and non-judgmental manner with local information that would help a Maltese consumer. Almost a quarter of the operators fell in the second category where the action was done without the consumer’s choice. For these operators (*n* = 12; 24%), four of these did not answer the statement, and the conversation was cut short. However, the first author received an email stating that a self-exclusion was placed on the account from all four operators. For the remaining eight operators, the customer service representative did not really answer the question and gave very vague answers such as affirming the statement or confirming that the situation must be difficult. Despite the answers not being ideal, for all these operators, the first author received an email stating that the account would be closed and that a self-exclusion was applied on the account. Bad practice was present for 11 operators (22%). For an operator to fall in this category, the response received to the statement would not be reflective of a responsible gambling operator and meant they did not provide support to an individual who admitted they had a gambling problem. These varied quite a bit. Examples include one operator highlighting that the choice is dependent on the consumer so there is nothing the operator can do. Another operator mentioned that the first author could have a bonus because current times are tough on everyone (supposedly referring to COVID-19 situation), and another customer service representative highlighted that it is only a gambling problem if an individual experienced it all the time and not periodically.

Given that the majority of the operators’ sample was seen as either showing good practice or self-excluding the first author without being given the choice, it was expected that most operators would not let the first author access their account again. Despite this, over one-fifth of the operators still allowed access to the account (*n* = 11; 22%), whereas 39 of the operators (78%) did not allow the first author to access the gambling account.

## Discussion

The aim of this study was to evaluate different online gambling operators and how they present their RG practices and tools in order to provide consumer protection. This study also replicated the study by Bonello and Griffiths (2017) to see if there were any changes in RG practices and use of RG tools given the current regulatory environment. This is important given the increasing use of RG tools to minimize harm to gamblers and the number of recent systematic reviews that have examined the efficacy of these tools (e.g., Bjørseth et al., 2021; Delfabbro & King, 2021; Drawson et al., 2017; Harris & Griffiths, 2017; Ladouceur et al., 2017; McMahon et al., 2019; Tanner et al., 2017).

Overall, there were differences noted from the previous 2017 study to the present study. These differences are presented in Table 2. The presence of an RG page was found for all, but two of the operators examined, and in one case, the RG page was not visible on the main page of the operator, where it is most needed. A notable change from the previous study was the reduction of promotional communication on the RG page. In the 2017 study, only six of the operators (12%) had no promotional communication on their RG page, whereas in this current study, 43 operators had no promotional material on the RG page (86%). Operators have a responsibility to their consumers to prevent gambling-related harm as much as possible (Monaghan, 2009), and providing a non-commercially inclined RG page is the least the operator may do. This also emphasizes that there is an element of responsible and fair practice by the operator, which is not only in accordance to the regulation and codes of conduct but increases customer retention which in turn aids to a profitable business model (Gainsbury et al., 2013).

**Table 2:** Comparison of the results from 2017 study and the present study

Criteria	2017	Current
RG dedicated page	*n* = 50; 100%	*n* = 48; 96%
- Statement of commitment to RG	*n* = 50; 100%	*n* = 48; 96%
- Information about gambling harm	*n* = 50; 100%	*n* = 48; 96%
- Mention of gambling help organization(s)	*n* = 42; 84%	*n* = 46; 92%
- Self-assessment	*n* = 32; 64%	*n* = 42; 84%
- Information about RG tools	*n* = 42; 84%	*n* = 46; 92%
- No promotional material	*n* = 6; 12%	*n* = 46; 92%
- Gambling blocking software links	*n* = 30; 60%	*n* = 27; 54%
Age checks	*n* = 34; 68%	*n* = 44; 88%
Link to RG in first email communication	*n* = 22; 44%	*n* = 21; 42%
Account history	*n* = 47; 94%	*n* = 48; 96%
Availability to limit setting	*n* = 45; 90%	*n* = 49; 98%
Cooling off functionality	*n* = 36; 72%	*n* = 48; 96%
Self-exclusion option	*n* = 43; 86%	*n* = 48; 96%
Customer services communication regarding limits	*n* = 30; 60%	*n* = 43; 86%
Customer services communication regarding RG breaks	*n* = 22; 44%	*n* = 39; 78%
Customer services communication for problem gamblers	*n* = 25; 50%	*n* = 31; 62%

Other than that, a high number of operators had sufficient information on their RG page to help the gambler make informed choice about the RG tools that they offer. Despite this, one of the suggestions would be to use easy to understand information about the RG tools and their offerings on the RG page. Some of the operators placed very technical, legal information on this page which was not always user-friendly. Having a more comprehensible representation of these RG tools would not only help the consumer refer to the page, but when displaying a positive attitude towards RG, and the tools, will help in normalizing these which will further help more in consumers using them (Procter et al., 2019). Gambling filtering software helps consumers that might be gambling too much to limit their gambling by reducing accessibility, even on websites that are not locally regulated; therefore, promoting such a tool would also help (Gamban, 2021).

Displaying information that the gambling product should be accessible for individuals above the legal age of 18 years increased in occurrence when compared to the 2017 study, from 68 (*n* = 34) to 88% (*n* = 44). Despite this, no operator realized that the account that the first author created did not contain real information; therefore, it might be easy for someone under the legal age to register and play. This can result in a major issue highlighted by Calado et al. (2017) because minors should not be allowed to gamble because gambling during adolescence may not only lead to problem gambling, but also addiction to other things (e.g., psychoactive substances). Similar to the 2017 study, almost all operators sent an email containing commercial information. This is understandable from a business perspective because it might encourage the consumer to play more, but less than half of the operators (42%; *n* = 21) only sent an email that contained some RG information or at least a link to the RG page. The most worrying aspect was that one operator’s link leading to the RG page was to commercial information, which could be either be a mistake or a non-responsible way of getting gamblers to play more.

Deposit limits are a good way of ensuring that consumers play within their limits, and voluntary deposit limits may encourage less churn and more sustainable revenue with the consumer through loyalty (Auer et al., 2019). These are also RG tools that appear to have some proven efficacy based on systematic literature reviews (e.g., Delfabbro & King, 2021; Harris & Griffiths, 2017). Although these tools may not be used frequently, their efficacy is still high, and consumers that set these limits gamble at sustainable levels (Gainsbury et al., 2020). The popularity of these tools may also be due to how the operator promotes such tools. The present study showed that less than half of the operators (40%; *n* = 20) offered the option to set a limit on the deposit money page, which might also influence how many gamblers use this limit more effectively.

Almost all the operators (98%, *n* = 49) offered RG tools such as limit-setting tools and short periods of taking a break from gambling. Most operators (96%, *n* = 48) offered the option for the consumer to have a break from gambling by using the self-exclusion tool. RG tools such as limit-setting and self-exclusion appear to be beneficial for the consumer and for the operator and are deemed useful by players (Griffiths et al., 2009) although proven efficacy of self-exclusion schemes is somewhat limited (Drawson et al., 2017; Tanner et al., 2017). A previous study found that online poker players mentioned that RG tools increase trust in the operator because it shows that the company has integrity (Wood & Griffiths, 2008). Regular consumers may set voluntary limits to prevent themselves from gambling too much (Hing et al., 2015). This is beneficial for the operator because it might increase the chance for the consumer never to go overboard and result in a sudden break from gambling. One factor which should be rectified is the offering of RG tools onsite. Five operators did not offer the consumer the option to self-exclude online, and this might not be the best RG approach because it might hinder consumers from taking a much-needed break from gambling.

The present study, as with the one in 2017, provides great insight because it involved active communication with the customer service representative. By using this methodology, the researchers were able to get a glimpse of the help a consumer would get first-hand from the operator. Although communication appears to have been better than the previous study, improvement is still needed. Online gambling is a very competitive business, and by focusing on consumer loyalty and investment, this would render the business successful (Gainsbury et al., 2013). Customer service interaction was the factor that differentiated the operators among them and showed which companies were really committed to RG. There may be a number of reasons why specific customer service interactions were not ideal. It could be that the operator is not taking RG that seriously, and therefore consumers highlighting that they might have an issue with gambling is not deemed as important enough to render an intervention. Another possible explanation could be that the customer service representatives were not sufficiently trained in RG, and therefore could not handle the customer contact properly.

### Limitations and Future Research

There are a number of limitations to the present study. Compared to the number of gambling websites worldwide, the number of websites examined was relatively small (although the present study did include the world’s most popular online gambling websites). In terms of replication of Bonello and Griffiths’ (2017) previous study of the world’s top 50 gambling websites, only 32 of these were still in operation at the time of the present study. Therefore, while the methodology was identical, the population sample was somewhat different in the present sample and may have influenced the findings. In relation to evaluating customer service interactions, only one interaction took place and may not necessarily have been representative of all customer services. Future studies should include more than a single interaction. Given that there is still improvement needed by gambling operators in the area of RG, future replication studies are needed along with a larger number of operators. Future research could also investigate whether there are differences in RG tool provision among gambling operators on different regulating jurisdictions. Other studies could also specifically examine how compliant the gambling operators are in relation to the jurisdictional gambling regulations.

### Conclusion

Online gambling is not a consumer behavior that will become obsolete, so prevention of harm and RG should be on the top of the agenda for any online gambling operator. Moreover, operators need to commit to RG, not only due to possible repercussions with the regulator, but also because it might cause customer disputes which would take up a lot of resources to resolve as well as increasing the potential for negative publicity (Gainsbury et al., 2013). The RG practices in the present study appear to have increased compared to Bonello and Griffiths’ previous study (2017), but there are still some areas that need to be improved. More specifically, these include better age checks at registration to prevent falsified identities and/or underage gambling, no promotional communication on the RG dedicated page, and more RG-oriented assistance when in communication with the online gambling operator representative.

### List of Online Gambling Websites

1. www.888ladies.com
2. www.bet-at-home.com
3. www.bet365.com
4. www.betfair.com
5. www.betsafe.com
6. www.betsson.com
7. www.betway.com
8. www.boomcasino.com
9. www.bwin.com
10. www.casinochan.com
11. www.casinogods.com
12. www.casinoeuro.com
13. www.casumo.com
14. www.cheekybingo.com
15. www.cherrycasino.com
16. www.comeon.com
17. www.coral.co.uk
18. www.costabingo.com
19. www.dreamvegas.com
20. www.energybet.com
21. www.eurogrand.com
22. www.expekt.com
23. www.fansbet.com
24. www.foxybingo.com
25. www.galacasino.com
26. www.jackpotcity.com
27. www.kingbilly.com
28. www.ladbrokes.com
29. www.leovegas.com
30. www.luckyredcasino.com
31. www.lvbet.com
32. www.mansioncasino.com
33. www.mrgreen.com
34. www.multilotto.com
35. www.netbet.com
36. www.nordicbet.com
37. www.paddypower.com
38. www.playjojo.com
39. www.pokerstars.com
40. www.queenvegas.com
41. www.redbet.com
42. www.riverbellecasino.com
43. www.rizk.com
44. www.sloty.com
45. www.spincasino.com
46. www.supercasino.com
47. www.stsbet.com
48. www.tipico.com
49. www.titanbet.com
50. www.williamhill.com
